# On the Rotation Method—The Construction of the First Gold Layers
*From William Herbst, Zahnarzt in Bremen.Translated from the *Correspondenz blatt fur Zahnartze,* by Anton J. Hecker, Dental Student, University of Maryland.


**Published:** 1886-06

**Authors:** 


### Editorial, Etc.

ON THE ROTATION METHOD—THE CONSTRUCTION OF THE FIRST GOLD LAYERS.*—As well understood, the construction of the first gold layers is quite hard work for a good many practitioners, especially when filling large cavities.

If the first layer of a filling, for instance, in approximal cavities in molars is not perfect, the whole filling is worthless.

Since November 1st, 1885, I experimented with all possible materials, for instance, tin foil, flannel, silk, spunk, rubber, etc., etc., to facilitate the construction of the first layer. Tin foil would be best, if it would not by its use take the adhering properties from the gold; still tin foil has got some very good properties, about which I will speak later on.

Purified cotton, I think, gives the best results. It must be thoroughly free of fat, and I think the preparation from C. Ash & Sons, the best one. The using of the cotton is very simple. After pressing a few slightly heated gold cylinders on the ground of the cavity, fill the same about one-half to two-thirds with cotton and press this one down with engine instruments, not too thick, for instance, No. 5. After removing the cotton again the gold has been pressed down around the floor and lower walls, equally and fitting as tight as possible.

When using cotton, a large quantity of gold should not be used for the forming of this first layer, less as when condensing directly with stone or steel instruments. Slightly heated gold pellets have then to be placed on this layer by the turning of hand instruments and then pressed down again in

---

*From William Herbst, Zahnarzt in Bremen.

Translated from the *Correspondenz blatt fur Zahnartze*, by Anton J. Hecker, Dental Student, University of Maryland.

the manner before described. After removing the cotton, the gold has to be condensed with stone or steel instruments and at last to be examined with the finest hand instruments if soft parts exist, which, if necessary have to be repaired with small pellets, so that the gold firmly attaches to the walls.

The advantages of this method are: 1st.—That the gold is perfectly tight on the base of the cavity. 2nd.—That the gold is equally pressed down and will not be any harder on the surface as on the base as it was pressed down without coming in direct contact with the instrument. The gold is hardened and pressed with rotating stone or steel instruments not before it lays perfectly tight in its place. The other layers can be treated with cotton or (as I described in my treatise) with stone or steel instruments.

